# Intravitreal Bevacizumab for Choroidal Neovascular Membrane in Macular Telangiectasia Type 2

**DOI:** 10.7759/cureus.40528

**Published:** 2023-06-16

**Authors:** Orlando G Gonzalez Martinez, Sofía C Ayala Rodríguez, Mariella Pappaterra-Rodriguez, Guillermo Requejo-Figueroa, Armando L Oliver

**Affiliations:** 1 Ophthalmology, University of Puerto Rico School of Medicine, Medical Sciences Campus, San Juan, USA; 2 School of Medicine, Ponce Health Sciences University, Ponce, USA

**Keywords:** right-angled vessels, case report, subretinal neovascularization, vascular endothelial growth factor, macular telangiectasia type 2

## Abstract

We report a case of macular telangiectasia type 2 with an associated choroidal neovascular membrane and its treatment. A 54-year-old female presented with a history of gradual vision loss in both eyes. A physical exam showed visual acuity of 20/40 in both eyes and significant metamorphopsia in the right eye. A fundus examination of the right eye was remarkable for right-angled vessels in the juxtafoveal region and subretinal fibrosis temporal to the fovea. A fundus examination of the left eye revealed intraretinal crystals in the juxtafoveal region and right-angled vessels. Optical coherence tomography and angiography confirmed the diagnosis of macular telangiectasia type 2 as well as the presence of a choroidal neovascular membrane in the right eye. The patient was treated with 18 intravitreal injections of anti-vascular endothelial growth factor agent in the right eye for two years, with five to six weeks between each treatment, which resulted in the membrane's stability. Our report suggests that anti-vascular endothelial growth factor therapy via intravitreal injection may be beneficial in treating choroidal neovascular membranes in patients with macular telangiectasia type 2.

## Introduction

Macular telangiectasia type 2 (MT2) is a retinopathy of unknown etiology characterized by abnormal vascular dilation of the capillaries in the juxtafoveal macula [1]. This disease was initially described by Gass as a distinct entity from Coats disease [2]. Past studies have shown that while its etiology was previously thought to arise from vascular disease, MT2 may be a neurodegenerative process, commencing with the loss of retinal Müller cells, leading to vascular occlusion, inflammatory changes, and retinal injury [1,3]. The disease tends to present bilaterally in middle-aged patients and does not have a predilection for either sex [1,3]. Large studies, including the MacTel project and the Beaver Dam Eye Study, have yielded conflicting findings regarding the prevalence of systemic diseases and their association with MT2 [4,5]. According to the MacTel project, patients diagnosed with MacTel demonstrated a higher prevalence of diabetes mellitus and hypertension [4]. However, the Beaver Dam Eye Study did not observe any significant associations with diabetes or hypertension [5].

Common complications of this disease process may include the formation of pseudo-lamellar macular holes, subretinal neovascularization, exudates, hemorrhages, or disciform scars [1,3]. Neovascular membranes are a complication that may occur; they often cause acute vision loss in patients if they arise near the fovea [6-8]. In MT2, these membranes tend to originate from the retinal vasculature instead of the choroidal vasculature, as is commonly seen in the late stages of age-related macular degeneration [3,6,9]. The early treatment of these neovascular processes may result in improved visual outcomes [3,9]. We present herein a case of MT2 with a juxtafoveal choroidal neovascular membrane treated with anti-vascular endothelial growth factor (anti-VEGF) monotherapy.

## Case presentation

A 54-year-old Hispanic female presented with gradual vision loss in both eyes (OU) for approximately three months. The patient had no pain, photophobia, ocular surgery, or trauma history. No relevant past medical history. Her family history was remarkable for diabetes mellitus type 2 in both of her parents.

Upon a comprehensive ophthalmic examination, her best-corrected visual acuity at the initial presentation was 20/40, OU. The intraocular pressure was 15 mmHg and 14 mmHg in the right eye (OD) and the left eye (OS), respectively. The pupils were both round and reactive to light, without any relative afferent defect. Both eyes had 1+ nuclear sclerosis cataracts. The patient refers to having significant metamorphopsia OD. Her color vision, assessed by the Ishihara color plate test, was unremarkable OU.

**Figure 1:**
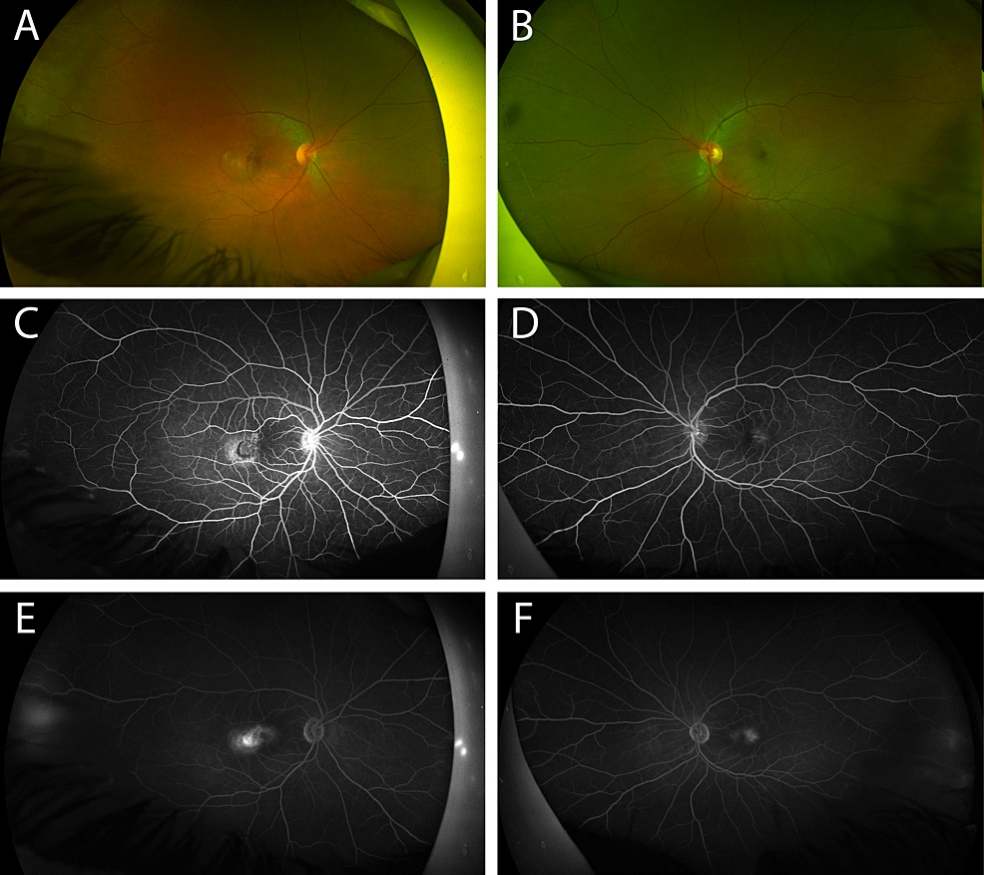
Fundus images of a patient with idiopathic macular telangiectasia type 2 Color fundus photograph of the right eye (A) reveals telangiectatic vessels and an area of subretinal fibrosis temporal to the fovea, consistent with a choroidal neovascular membrane while the left fundus photograph (B) reveals intraretinal crystals in the juxtafoveal region and right-angled vessels. In the right eye, the early and late phases of a fluorescein angiogram (C and E, respectively) reveal staining within the observed area of subretinal fibrosis and associated leakage in the temporal aspect of the lesion, consistent with a choroidal neovascular membrane. In the left eye, the early and late phases of the fluorescein angiogram (D and F, respectively) reveal leakage temporal to the fovea that evolves into leakage of the entire foveal region.

**Figure 2:**
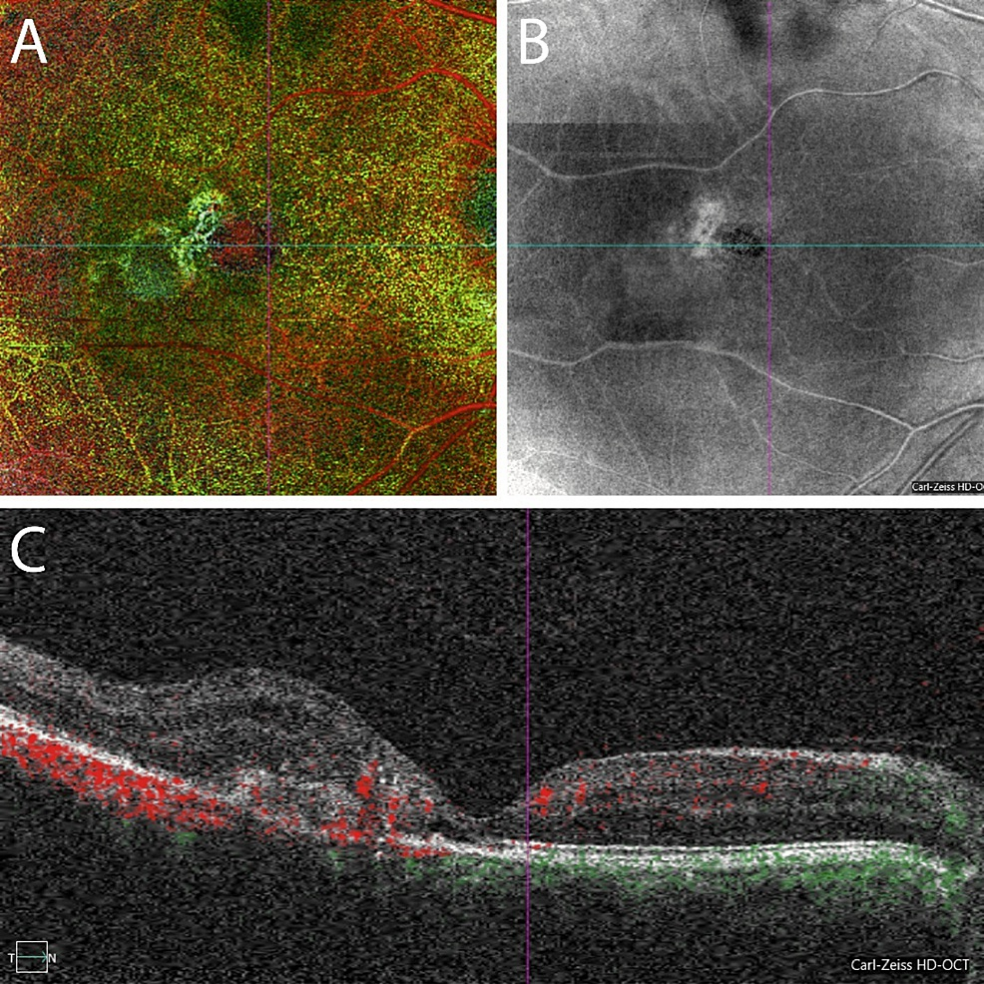
Right eye en-face color depth encoded optical coherence tomography angiography (A), structural en-face intensity image (B), and corresponding B-scan (C) of the right eye OCT angiography of the right eye. The multilayer macular view (A) and the OCT photograph (B) reveal the presence of neovascular vessels and subretinal fibrosis. Spectral-domain OCT images with OCTA overlay (C) of the right eye reveal a loss of regular foveal depression and the presence of subretinal hyperreflective material, as well as evidence of intraretinal and subretinal fluid leading to central thickening, and localize the neovascular vessels as extending from the choriocapillaris through the inner plexiform layer.

A dilated fundus examination of the OD revealed right-angled vessels in the juxtafoveal region and subretinal fibrosis temporal to the fovea (Figure 1A). A dilated fundus exam of the OS revealed intraretinal crystals in the juxtafoveal region and right-angled vessels (Figure 1B). A fluorescein angiography of the OD revealed staining within the observed area of subretinal fibrosis and associated leakage in the temporal aspect of the lesion, consistent with a choroidal neovascular membrane OD (Figures 1C, 1E) while fluorescein angiography of the OS revealed early leakage temporal to the fovea that developed into parafoveal late leakage, consistent with a diagnosis of MT2 (Figures 1D, 1F). An OCT angiography showed a neovascular membrane present below the retinal pigment epithelium (RPE), with neovascular vessels extending from the choriocapillaris through the inner plexiform layer (Figures 2A, 2B). Spectral-domain optical coherence tomography (OCT) images of the OD revealed a loss of regular foveal depression and the presence of subretinal hyperreflective material, as well as evidence of intraretinal and subretinal fluid leading to central thickening (Figure 2C) while in the OS, there was a hyporeflective, fluid-like space.

A clinical diagnosis of MT2 with associated choroidal neovascular membrane OD was made. Anti-VEGF injections, including bevacizumab and aflibercept, were recommended for the choroidal neovascular membrane in her right eye. Treatment began one week after the diagnosis, with re-evaluation and retreatment every four to six weeks.

**Figure 3:**
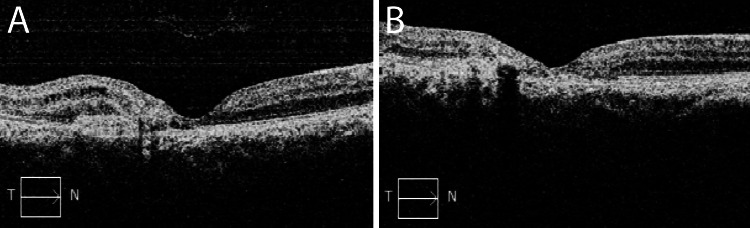
Spectral-domain OCT images of the right eye at presentation and after two years of therapy Spectral-domain OCT images of the right eye at presentation (A), revealing loss of foveal contour, presence of subretinal hyperreflective material, as well as intraretinal and subretinal fluid leading to foveal thickening. After two years of therapy (B), the image shows a marked improvement in the intraretinal and subretinal fluid and an enhanced appearance of the foveal depression.

Over the course of two years, the patient received a total of 18 injections of anti-VEGF medication OD. Aflibercept was used three times, while bevacizumab was used 15 times. She maintained a visual acuity of 20/30 OD and 20/25 OS. The patient stated that her vision had markedly improved after treatment. No symptoms were present at the most recent examination. The fundus findings have been stable since commencing treatment. Her latest OCT revealed intraretinal cystic changes, mild macular thickening, the absence of subretinal fluid, and a decrease in the size of the choroidal neovascular membrane OD and intraretinal cystic changes without macular thickening OS (Figure 3).

## Discussion

The etiology of MT2 is unclear. Initially, Yannuzzi et al. proposed an etiology that consists of vascular insufficiency leading to hypoperfusion, retinal tissue insults, and subsequent atrophy [1]. Regardless, the leading theory proposed by recent studies involves a neurodegenerative etiology caused by the loss of retinal Müller cells, which leads to the abnormal dilation of blood vessels [10]. MT2 is generally divided into two stages. The non-proliferative stage is characterized by progressive foveal thinning and edema while the proliferative stage is characterized by the formation of subretinal neovascularization [6,7].

Subretinal neovascularization is one of the main causes of vision loss in MT2 [11]. Studies suggest that subretinal neovascularization in MT2 originates from the retinal blood vessels resulting in an intraretinal anastomosis, as reported in Soheilian et al.’s study with indocyanine green angiography [12]. However, retinal-choroidal anastomosis has also been observed in many patients with MT2 [1,6,13,14]. Any disruption of the RPE may predispose to choroidal neovascularization [1,3]. Typically, neovascular membranes that arise in patients with MT2 tend to develop temporal to the fovea [3,7]. The neovascular vessels of which these membranes are composed most commonly appear to originate from the retinal vasculature but may extend themselves into the subretinal space and develop chorioretinal shunts [1,3,7]. In our patient, the vessels seemed to originate in the choroidal vasculature and the vessels extend into the intraretinal tissue as observed in Figure 2. The presence of this unusual membrane might be an indication that MT2 could also involve the choroid to some extent.

The management of neovascular membranes generally involves treatment with intravitreal anti-VEGF agents [6,7,9]. The chronicity of choroidal neovascular membranes may also play a part in the visual prognosis, as heavily cicatricial neovascular membranes will respond less favorably to treatment than those that are treated in the early stages of neovascularization [7]. Sandhu et al. found that their cohort did not have improved visual acuity in response to intravitreal anti-VEGF therapy but did find that the neovascular membranes regressed [15]. Moreover, Karagiannis et al. reported a case in which monthly injections of ranibizumab were effective in a patient with proliferative MT2 [16]. The development of a cell-based drug delivery system for ciliary neurotrophic factor (CNTF) holds great potential as a treatment modality [17]. In Phase 2 trials, this treatment demonstrated a substantial reduction in the progression of retinal degeneration [17].

The early diagnosis of neovascular changes on OCT may lead to improved visual outcomes, paying particular attention to the earliest signs noted on OCT, which include temporal widening of the foveal pit [14,18]. The patient's visual improvement may be attributed to the relatively early detection of the neovascular membrane during its active phase of neovascular proliferation [14]. A diagnosis of MT2 commonly occurs in middle-aged patients who begin to lose their vision [1]. However, the development of a choroidal neovascular membrane near the fovea may cause a sudden onset of vision loss, possibly leading to an earlier diagnosis [7,9]. Disease severity may be associated with age of onset, with earlier disease occurrence having a worse prognosis [3]. When evaluating visual acuity using conventional methods, the progression of MT2 seems to be gradual [19]. Studies have indicated that microperimetry testing is more effective in identifying functional deterioration, demonstrating greater sensitivity in detecting changes in visual function [19]. Nonetheless, knowledge of the presenting symptoms, morphologic alterations on imaging, and current treatments allow for earlier accurate diagnosis and potentially better prognosis.

## Conclusions

Our case demonstrates that anti-VEGF therapy may be beneficial in treating choroidal neovascular membranes in patients with MT2. The early diagnosis of macular telangiectasia may be more common in patients who develop neovascular membranes near the fovea.
